# Occupational Stress and Its Effect on Health Status Among Karnataka State Road Transport Corporation (KSRTC) Bus Drivers: A Cross-Sectional Study

**DOI:** 10.7759/cureus.70336

**Published:** 2024-09-27

**Authors:** Varun Rajan, Pradeep TS, Muninarayana Chandrappa

**Affiliations:** 1 Community Medicine, Sri Devaraj Urs Medical College, Sri Devaraj Urs Academy of Higher Education and Research, Kolar, IND

**Keywords:** bus drivers, health status, occupational stress, public transport workers, work place stress

## Abstract

Background: Occupational stress is a critical health concern across various professions, often linked to adverse health outcomes and increased risk-taking behaviors. Bus drivers are especially vulnerable due to prolonged shifts, exposure to environmental pollutants, and higher rates of sickness absenteeism. This study aimed to assess the prevalence of occupational stress among Karnataka State Road Transport Corporation (KSRTC) bus drivers and the factors contributed. In addition, it explores the relationship between job-related stress and overall health status, providing insights into the occupational challenges faced by this workforce.

Materials and methods: A cross-sectional study was conducted over two years from June 1, 2022, to June 30, 2024, at the KSRTC depot in Kolar, serving urban and rural areas. A representative sample of 235 bus drivers was selected from a total of 654 using simple random sampling. Socio-demographic details, including age, gender, education, religion, family type, and per capita income, were collected using a pre-tested semi-structured self-administered questionnaire. Stress levels were assessed using the American Institute of Stress (AIS) questionnaire, a widely used tool for evaluating various stress dimensions. Data were collected through interviews, entered into Excel, and subsequently analyzed using IBM SPSS Statistics for Windows, Version 22 (Released 2013; IBM Corp., Armonk, New York, United States).

Results: The study revealed that 65% of KSRTC bus drivers experienced moderate to severe stress. Significant factors associated with occupational stress included urban residency, divorce, chronic alcohol consumption, current tobacco chewing, hypertension, higher BMI, long working hours, and high waist-hip ratio. Binary logistic regression showed urban residents had higher stress odds than rural residents (OR=1.27, p=0.03). Divorced individuals and chronic alcoholics also had higher odds of stress (OR=1.24, p=0.05 and OR=1.33, p=0.04, respectively). Stress was significantly associated with hypertension (OR=1.40, p=0.01), obesity (OR=1.30, p=0.03), morbid obesity (OR=1.47, p=0.01), high-risk waist-hip ratios (OR=1.42, p=0.01), and longer working hours (>12 hours, OR=1.30, p=0.03). Health assessments revealed a high prevalence of hypertension (39%) and obesity (65%).

Conclusion: The study highlights a high prevalence of occupational stress among KSRTC bus drivers in Kolar, with 65% experiencing moderate to severe stress. Factors such as urban residency, lifestyle habits, and physiological parameters significantly contribute to stress. These findings underscore the need for targeted health promotion programs and policy changes to enhance the health of essential workers and improve road safety.

## Introduction

Occupational stress, a significant challenge for workers, particularly bus drivers, arises from demanding work conditions like long shifts, heavy workloads, and poor physical environments. These stressors, including traffic congestion, customer conflicts, and rigid timetables, contribute to health issues that affect overall well-being and productivity. Bus drivers, essential to the reliability of transportation services, often face early retirement due to stress-induced disabilities. Their well-being is vital for ensuring the safety and satisfaction of passengers [1,2].

Sedentary behavior, characterized by low energy expenditure while sitting or lying down, is prevalent among working-age adults, especially in the transport sector, and is linked to a heightened risk of cardiovascular diseases, mortality, and diabetes, regardless of physical activity during leisure time. Stress, as classified by the WHO into acute, episodic, and chronic types, exacerbates these risks, with acute stress causing short-term symptoms, episodic stress leading to frequent health issues, and chronic stress contributing to long-term conditions like hypertension and heart disease [3,4].

Bus drivers, especially those on long-haul routes, often face sleep disturbances due to inadequate resting conditions provided by employers. This lack of proper facilities leads to poor sleep, heightening stress, anxiety, and concentration issues, which in turn increase the risk of accidents and injuries, endangering both drivers and passengers [5].

The demanding work schedules significantly increase the stress levels among drivers, who often feel pressured by tight deadlines. The working conditions for bus personnel involve not only time constraints but also ergonomic challenges and security concerns. This involves making workplace adjustments, implementing administrative changes, and educating drivers. Addressing factors like prolonged sitting, whole-body vibration, and the ergonomic fit between drivers and their environment, including vehicle seats, are essential for creating a safer driving experience [6,7].

Bus drivers, especially those on long-haul routes, often face sleep disturbances due to inadequate resting conditions provided by employers. This lack of proper facilities leads to poor sleep, heightening stress, anxiety, and concentration issues, which in turn increase the risk of accidents and injuries, endangering both drivers and passengers. The burden of stress among bus drivers has been well documented in various studies across different countries. In Iran, Hokmabadi et al. (2018) reported an exceptionally high prevalence of job stress at 97% among bus drivers [8]. Similarly, Bathija et al. (2014) found that 80% of Indian bus drivers experienced significant stress levels [9]. In a more recent study, Useche et al. (2022) noted that 40% of bus drivers in Colombia were affected by stress, highlighting the global nature of this occupational health concern [10].

This study highlights the critical gap in understanding stress and health issues among bus drivers in organized sectors such as Karnataka State Road Transport Corporation (KSRTC). The findings highlight the need for targeted health interventions and policies to mitigate occupational health risks, with a focus on the prevalence and determinants of occupational stress among KSRTC bus drivers in Kolar, Southern India. The study was done with the objective of determining occupational stress levels and their association with job-related factors among KSRTC bus drivers in Kolar.

## Materials and methods

The present is a cross-sectional study conducted for a period of two years, from June 1, 2022, to July 30, 2024. Kolar District comprises six taluks: Mulbagal, Kolar, Bangarapet, Malur, Srinivaspura, and Kolar Gold Fields (KGF) [11]. The depot employs 729 individuals, categorized into outdoor workers, such as bus drivers (654), conductors, mechanics, and housekeeping staff, and indoor workers, comprising administrative personnel based in office buildings within the depot premises. The study included KSRTC bus drivers currently employed with at least one year of driving experience, aged 21 to 60 years. Drivers absent for more than six months for any reason, including health, were excluded. The sample size was determined based on an 83.3% prevalence of stress from a prior study, using a 95% confidence interval (Z_α_=1.96) and 5% relative precision (d=0.05). This calculation yielded a minimum required sample size of 216, which, after accounting for a 10% non-response rate, was adjusted to a final sample size of 240 [12].

The clinicodemographic profile was assessed using a pretested, structured questionnaire. Stress levels were evaluated with the American Institute of Stress (AIS) questionnaire, which covers work and personal life stressors, coping mechanisms, and overall well-being. Participants rated their stress on a scale from 1 to 5, with part A focusing on job-related feelings and part B on general satisfaction and stress over the past year. Scores range from 0-40: 0-15 (relatively calm), 16-20 (mild stress), 21-25 (moderate stress), 26-30 (severe stress), and 31-40 (potentially dangerous stress levels [13].

**Figure 1:**
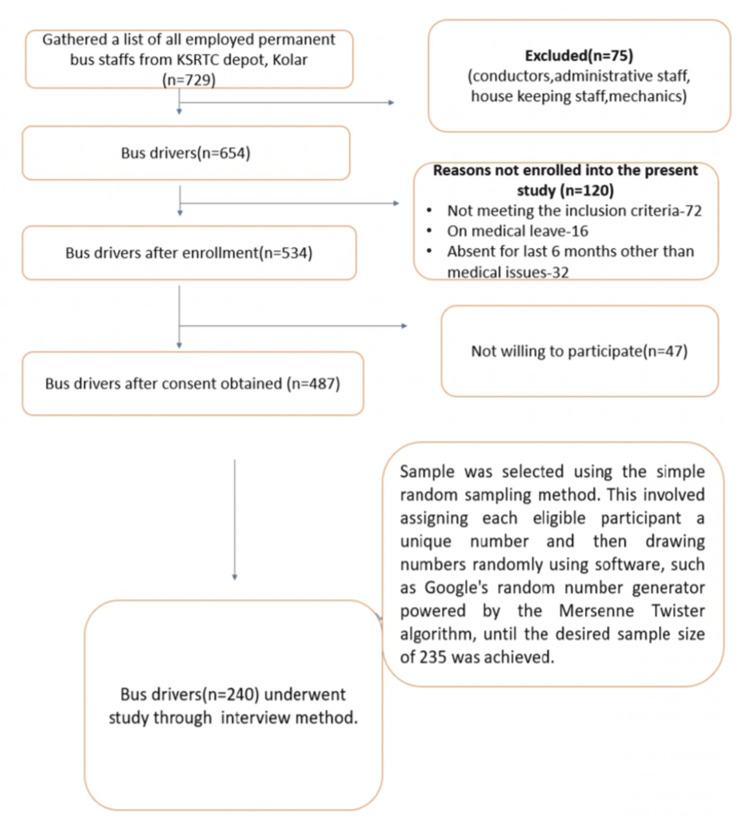
Summary of flow of participants in sampling scheme for the study KSRTC: Karnataka State Road Transport Corporation

A sampling procedure was implemented to gather a list of permanently employed staff from the competing authority of the KSRTC bus depot in Kolar. At that time, the depot housed 654 bus drivers. To obtain a representative sample, 240 individuals were required. This sample was selected using the simple random sampling method, facilitated by a software Google random generator powered by the Mersenne Twister algorithm [14]. Each selected participant was subjected to a 10-minute interview session as part of the data collection process (Figure 1).

Socio-demographic details such as age, gender, education, religion, family type, and per capita income were collected through a pre-tested, semi-structured, self-administered questionnaire. The study also gathered data on substance abuse alongside coexisting health conditions like diabetes, hypertension, and coronary artery disease. Moreover, participants' anthropometric measurements, including waist-to-hip ratio, were recorded.

In this study, key lifestyle behaviors and health conditions were defined to assess their prevalence and impact among participants. Cigarette/beedi smoking was categorized into never smokers (those who have never smoked), current smokers (with details on the number of cigarettes or beedis smoked per day and duration in years), and ex-smokers (with the total duration of smoking recorded). Chewable tobacco use was similarly classified into never chewers, current chewers (with a frequency of use per day and duration in years), and ex-chewers (with the duration of previous use noted). Alcohol consumption was categorized into never-drinkers, alcohol drinkers (with data on the number of years of consumption, frequency per week, and average number of standard drinks consumed per week), and chronic alcoholics (those who drink daily, particularly in the morning for five years) [15]. Hypertension and diabetes were identified based on OP card records maintained by participants, following American Heart Association criteria for hypertension and specific blood glucose or HbA1c levels for diabetes, including those on medication [16]. Work shifts were categorized by day, night, or rotating schedules, and work experience by total years as a bus driver. Marital status was classified into unmarried, married, widowed, divorced, or separated, with education levels ranging from illiterate to professional degrees [17]. The waist-hip ratio was categorized into low, moderate, and high-risk groups [18]. Socioeconomic status was assessed using the Modified BG Prasad Classification (2023) into five income-based classes [19].

Ethical approval was obtained from the Institutional Ethics Committee of Sri Devaraj Urs Academy of Higher Education and Research via reference no. SDUMC/KLR/IEC/242/2022-23. The written informed consent form was obtained from the KSRTC depot manager and participants.

After thorough cleaning, the data were coded in Excel and analyzed using IBM SPSS Statistics for Windows, Version 22 (Released 2013; IBM Corp., Armonk, New York, United States). Descriptive statistics (percentages, mean±standard deviation) were used for variables like religion, BMI categories, and age. The chi-square test assessed the association between categorical variables (e.g., religion, education) and occupational stress severity, while univariate and multivariate logistic regression provided crude and adjusted odds ratios with 95% confidence intervals to predict stress. A p-value of less than 0.05 was considered statistically significant for the chi-square test, and 95% confidence intervals excluding the null value were deemed significant for both crude and adjusted odds ratios.

## Results

The clinical sociodemographic profile of KSRTC bus drivers in the Kolar bus depot (n=240) exhibits that the majority are aged 36-50 years (170 (70.8%)) and reside in urban areas (151 (63%)). Most drivers have 6-10 years of work experience (95 (39.6%)), and a significant portion is married (186 (78%)). A large majority belong to the upper middle class (215 (89.6%)) according to Modified BG Prasad classification September 2023, with 193 drivers (80%) following a mixed diet. Smoking is prevalent, with 104 drivers (43%) being current smokers, while 83 (35%) currently chew tobacco. Alcohol consumption is reported by 102 drivers (43%), and 57 drivers (24%) have diabetes, with 93 (39%) having hypertension. Work shifts were predominantly flexible, with 181 drivers (75%) working either day or night shifts, 39 drivers (16%) working night shifts, and 20 drivers (8%) working only during the day (Table 1).

**Table 1:** Distribution of KSRTC bus drivers according to clinic sociodemographic profile in Kolar bus depot, Karnataka (n=240) KSRTC: Karnataka State Road Transport Corporation

Variables		Frequency	Percentage
Age in years	20-35	50	20.8%
	36-50	170	70.8%
	51-65	20	8.3%
Area of residency	Urban	151	63%
	Rural	89	37%
Work experience (years)	0-5	33	13.8%
	6-10	95	39.6%
	11-15	84	35%
	16-20	17	7.1%
	21-25	11	4.6%
Marital status	Unmarried	30	13%
	Married	186	78%
	Others (divorced, separated, widower)	24	9%
Socioeconomic class (according to modified BG Prasad classification October 2023)	Upper class (>Rs 8822)	10	4.2%
	Upper middle class (Rs 4411-8821)	215	89.6%
	Middle class (Rs 2647-4410)	15	6.3%
Diet	Vegetarian	47	20%
	Mixed	193	80%
Smoking cigarette	Never smoked	91	38%
	Current smoker	104	43%
	Smoked before, now quit	45	19%
Chewable tobacco	Never chewed	113	47%
	Current chewer	83	35%
	Chewed before, now quit	44	18%
Alcohol drinking	Never	133	55%
	Alcohol drinker	102	43%
	Chronic alcoholic	5	2%
History of diabetes	Yes	57	24%
	No	183	76%
History of hypertension	Yes	93	39%
	No	147	61%
Waist hip circumference ratio (males)	Lower risk (<0.95)	229	95.4%
	Moderate risk (0.96-1)	9	3.8%
	High risk (>1)	2	0.8%
Working hours	<12 hours	55	23%
	>12 hours	185	77%
Work shift	Day	20	8%
	Night	39	16%
	Either	181	75%

In this study, the occupational stress levels among KSRTC bus drivers were assessed using the AIS questionnaire, revealing that a significant proportion of the participants experienced varying levels of stress. Out of the 240 drivers interviewed, a majority of 157 drivers (65%) were found to have moderate stress levels, while 83 drivers (35%) exhibited severe stress (Table 2).

**Table 2:** Distribution of KSRTC bus drivers according to occupational stress measured using American Institute of stress questionnaire (n=240) KSRTC: Karnataka State Road Transport Corporation

Occupational stress score	Score	Levels	Frequency	Percentage
	0-15	Relatively calm	0	0%
	16-20	Mild stress	0	0%
	21-25	Moderate level stress	157	65%
	26-30	Severe level of stress	83	35%
	31-40	Potentially danger	0	0%

The present study reveals significant associations between socio-demographic and lifestyle factors and occupational stress among KSRTC bus drivers in Kolar. Drivers in the upper socioeconomic class (p=0.014), those on a mixed diet (p=0.044), chronic alcohol drinkers (p=0.018), and individuals with a normal BMI (p=0.033) were significantly associated with severe stress. These findings highlight the role of socioeconomic status, dietary habits, alcohol use, and BMI in influencing occupational stress levels in this population (p<0.05) (Table 3).

**Table 3:** Association of clinicosocio-demographic and lifestyle variables with occupational stress levels among KSRTC bus drivers in Kolar (n=240) KSRTC: Karnataka State Road Transport Corporation

Variables			Moderate stress (157 (65%))	Severe stress (83 (35%))	Chi-square value (p-value)
Age in years	20-35		36 (72%)	14 (28%)	χ²=1.227, p=0.541
	36-50		108 (63.5%)	62 (36.5%)	
	51-65		13 (63.5%)	7 (35%)	
Area of residency	Urban		101 (66.9%)	50 (33.1%)	χ²=0.389, p=0.533
	Rural		56 (62.9%)	33 (37.1%)	
Work experience (years)	0-5		21 (64%)	12 (36%)	χ²=3.27, p=0.514
	6-10		57 (60%)	38 (40%)	
	11-15		58 (69%)	26 (31%)	
	16-20		12 (70%)	5 (30%)	
	21-25		9 (81%)	2 (19%)	
Marital status	Unmarried		23 (76%)	7 (24%)	χ²=3.590, p=0.464
	Married		118 (64%)	68 (36%)	
	Others (divorced, separated, widower)		16 (62%)	8 (38%)	
Socioeconomic class (according to modified BG Prasad classification (October) 2023)	Upper class (>Rs 8822)		6 (60%)	4 (40%)	χ²=8.503, p=0.014^*^
	Upper middle class (Rs 4411-8821)		136 (64%)	79 (36%)	
	Middle class (Rs 2647-4410)		15 (100%)	0 (0%)	
Diet	Vegetarian		31 (66%)	16 (34%)	χ²=6.261, p=0.044^*^
	Mixed		126 (66%)	67 (34%)	
Smoking cigarette	Never smoked		66 (73%)	25 (27%)	χ²=8.17, p=0.086
	Current smoker		62 (60%)	42 (40%)	
	Smoked before, now quit		29 (64%)	16 (36%)	
Chewable tobacco	Never chewed		78 (69%)	35 (31%)	χ²=2.293, p=0.318
	Current chewer		49 (59%)	34 (41%)	
	Chewed before, now quit		30 (68%)	14 (32%)	
Alcohol drinking	Never		97 (73%)	36 (27%)	χ²=8.047, p=0.018^*^
	Alcohol drinker		58 (56%)	44 (44%)	
	Chronic alcoholic		2 (40%)	3 (60%)	
History of diabetes	Yes		38 (66%)	19 (33%)	χ²=0.052, p=0.820
	No		119 (65%)	64 (35%)	
History of hypertension	Yes		64 (69%)	29 (31%)	χ²^=^0.776, p=0.378
	No		93 (63%)	54 (67%)	
Waist hip circumference ratio (males)	Lower risk (<0.95)		153 (67%)	76 (33%)	χ²=4.502, p=0.105
	Moderate risk (0.96-1)		3 (33%)	6 (67%)	
	High risk (>1)		1 (50%)	1 (50%)	
Working hours	<12 hours		17 (85%)	3 (15%)	χ^2^=0.10, p=0.752
	>12 hours		23 (59%)	16 (41%)	
Work shift	Day		17 (85%)	3 (15%)	χ²=4.15, p=0.125
	Night		23 (59%)	16 (41%)	
	Either		117 (65%)	64 (35%)	
BMI category	Normal		6 (36%)	11 (64%)	χ²=8.716, p=0.033^*^
	Overweight		17 (59%)	12 (42%)	
	Obese		106 (69%)	49 (32%)	
	Morbidly obese		28 (72%)	11 (28%)	
^*^p<0.05, statistically significant at 95% confidence interval					

Binary logistic regression was carried out to find out the association between occupational stress and socio-demographic and lifestyle factors. Urban residency (adjusted OR: 1.27, p=0.03), longer work hours (adjusted OR: 1.30, p=0.03), 21-25 years of work experience (adjusted OR: 1.47, p=0.03), chronic alcohol consumption (adjusted OR: 1.33, p=0.04), hypertension (adjusted OR: 1.40, p=0.01), morbid obesity (adjusted OR: 1.47, p=0.01), and night shift work (adjusted OR: 1.42, p=0.01) were all significantly associated with increased occupational stress, each showing an odds ratio greater than 1 (Table 4).

**Table 4:** Binary logistic regression to study the association of occupational stress with socio demographic characteristics among KSRTC bus drivers Kolar depot, Karnataka (n=240) KSRTC: Karnataka State Road Transport Corporation

Covariates		Crude OR	95% CI	p-value	Adjusted OR	95% CI (adjusted)	p-value
Area of residence	Rural (ref)	1.0	-	-	1.0	-	-
	Urban	1.30	1.05-1.61	0.01	1.27	1.02-1.58	0.03
Work experience in years	0-5	1.0	-	-	1.0	-	-
	6-10	1.12	0.91-1.38	0.27	1.10	0.89-1.35	0.42
	11-15	1.24	1.01-1.52	0.04	1.19	0.97-1.46	0.13
	16-20	1.38	1.12-1.69	0.02	1.32	1.07-1.63	0.07
	21-25	1.53	1.24-1.89	0.01	1.47	1.19-1.81	0.03
Marital status	Unmarried (ref)	1.0	-	-	1.0	-	-
	Married	1.18	0.95-1.46	0.14	1.13	0.91-1.40	0.27
	Divorced	1.29	1.04-1.60	0.02	1.24	1.00-1.53	0.05
	others	1.11	0.89-1.38	0.35	1.06	0.85-1.32	0.54
Socioeconomic class	Middle	1.0	-	-	1.0	-	-
	Upper Middle	1.15	0.93-1.42	0.22	1.11	0.90-1.37	0.37
	Upper	1.19	0.96-1.47	0.12	1.14	0.92-1.41	0.28
Education	Primary school	1.0	-	-	1.0	-	-
	High school	1.09	0.87-1.36	0.43	1.04	0.83-1.30	0.63
	PUC	1.18	0.95-1.47	0.16	1.13	0.91-1.40	0.29
	Diploma	1.26	1.02-1.55	0.03	1.21	0.98-1.49	0.08
Diet	Vegetarian(ref)	1.0	-	-	1.0	-	-
	Mixed	1.09	0.88-1.35	0.54	1.07	0.86-1.33	0.65
Smoking	Non-smoker (ref)	1.0	-	-	1.0	-	-
	Smoked before, now quit	1.15	0.93-1.42	0.24	1.11	0.89-1.38	0.41
	Current smoker	1.30	1.05-1.60	0.04	1.25	1.01-1.54	0.08
Chewable tobacco	Never Chewed	1.0	-	-	1.0	-	-
	Chewed before, now quit	1.20	0.98-1.47	0.06	1.15	0.93-1.42	0.23
	Current Chewer	1.35	1.10-1.65	0.01	1.30	1.06-1.59	0.03
Alcohol consumption	Never	1.0	-	-	1.0	-	-
	Alcohol drinker	1.25	1.02-1.53	0.03	1.20	0.97-1.48	0.08
	Chronic Alcoholic	1.40	1.15-1.70	0.01	1.33	1.09-1.62	0.04
History of diabetes	No (ref)	1.0	-	-	1.0	-	-
	Yes	1.30	1.08-1.57	0.02	1.25	1.03-1.52	0.05
History of hypertension	No (ref)	1.0	-	-	1.0	-	-
	Yes	1.45	1.20-1.75	0.00	1.40	1.15-1.70	0.01
BMI categories	Normal (ref)	1.0	-	-	1.0	-	-
	Overweight	1.20	0.97-1.48	0.09	1.15	0.93-1.42	0.26
	Obese	1.35	1.10-1.66	0.01	1.30	1.06-1.59	0.03
	Morbidly obese	1.55	1.26-1.90	0.00	1.47	1.19-1.81	0.01
Waist-hip circumference ratio	Low risk (ref)	1.0	-	-	1.0	-	-
	Moderate risk	1.25	1.02-1.53	0.03	1.20	0.98-1.47	0.07
	High risk	1.50	1.22-1.84	0.00	1.42	1.16-1.74	0.01
Working hours per week	<12 hours	1.0	-	-	1.0	-	-
	>12 hours	1.35	1.10-1.66	0.01	1.30	1.06-1.59	0.03
Work shift	Day shift (ref)	1.0	-	-	1.0	-	-
	Either shift	1.20	0.97-1.48	0.08	1.15	0.93-1.42	0.24
	Night shift	1.50	1.22-1.84	0.00	1.42	1.16-1.74	0.01

## Discussion

In this cross-sectional study conducted over two years (June 2022 to July 2024) among 240 KSRTC bus drivers in India, we assessed the prevalence and predictors of job stress. The study identified several key factors associated with elevated stress levels, such as urban residency, long work hours, certain work experiences, marital status, and lifestyle behaviors like smoking, tobacco chewing, and alcohol consumption. Comorbidities, including hypertension, diabetes, and obesity, along with night shifts, also emerged as significant predictors of occupational stress through multivariable analysis. This highlights the multifaceted contributors to stress in this occupational group without reiterating detailed findings from the results section.

Our study showed a prevalence of alcohol consumption of 43%, similar to the study done by Cunradi et al. in San Francisco, which reported a prevalence of 42%, while Kaul et al.'s study in India showed a prevalence of 83%, possibly due to cultural differences and variations in alcohol availability and social acceptance between the regions [20,21]. The current study revealed that 43% of the participants were current smokers, similar to the 54% prevalence reported by Prabhu et al. in India in 2015, while Useche et al.'s 2017 study in Colombia found a lower prevalence of 27.8%, possibly due to differences in tobacco regulations and cultural attitudes toward smoking in these regions [22,23]. The prevalence of hypertension in our study was 39%; this finding is comparable to the prevalence reported by Rao et al. (36%) among bus drivers in India but lower than the prevalence reported by Modjadji et al. (57%) in South Africa [24,25]. The present study revealed a high prevalence of occupational stress among KSRTC bus drivers, with 65% experiencing moderate stress and 35% experiencing severe stress, comparable to the findings of Bathija et al. (80%), Mohsen and Hakim (83.2%), and Rathi et al. (58.3%) among bus drivers in India and other countries. These variations could be attributed to differences in working conditions, job demands, and support systems across different regions and studies [9,12,15].

In our study, severe stress was significantly associated with a mixed diet among KSRTC bus drivers (χ²=6.261, df=2, p=0.044). This aligns with Rao et al.'s findings in India, which also reported a significant association between dietary habits and stress levels in bus drivers [24]. Moreover, our study showed a significant relationship between stress and alcohol consumption, particularly among chronic alcoholics (χ²=8.047, df=2, p=0.018), substantiating the findings of Kaul et al. in 2019 [21]. Similarly, we found a significant association between stress levels and BMI categories among KSRTC bus drivers (χ²=8.716, df=3, p=0.033), consistent with Joshi et al.'s 2013 study, which also highlighted a link between BMI and stress among Indian bus drivers [26].

In our univariate analysis, socioeconomic status, BMI, alcohol consumption, and diet were significantly associated with stress. To identify key risk factors, we performed binary logistic regression, including variables significant at the 0.2% level in the univariate analysis. Binary logistic regression analysis revealed several significant risk factors for stress among KSRTC bus drivers. Urban residents had 1.27 times higher odds of experiencing stress compared to rural residents (OR=1.27, p=0.03), consistent with Adedokun et al. (2019), who observed a similar trend among urban bus drivers in South Africa [27]. Divorced individuals showed 1.24 times higher odds of stress compared to unmarried individuals (OR=1.24, p=0.05) (OR=1.24, p=0.05). Similarly, current tobacco chewers had 1.30 times higher odds of stress compared to non-chewers (OR=1.30, p=0.03), similar to the findings by Parashari et al. (2017) among Indian drivers [28]. Chronic alcoholics were 1.33 times more likely to experience stress than non-drinkers (OR=1.33, p=0.04), consistent with Kaul et al. (2019), who also found higher stress levels among chronic alcoholics in India [21]. Individuals with hypertension had 1.40 times higher odds of stress compared to those without hypertension (OR=1.40, p=0.01), reflecting Walvekar et al.'s (2021) findings in India [29]. Obese and morbidly obese participants had 1.30 and 1.47 times higher odds of stress, respectively, compared to those with a normal BMI (OR=1.30, p=0.03; OR=1.47, p=0.01), as supported by Rao et al. (2015) [24]. Moreover, bus drivers working over 12 hours per week had 1.30 times higher odds of stress compared to those with shorter hours (OR=1.30, p=0.03), consistent with Sebastin's (2019) findings [30]. Our study found a high prevalence of overweight (65%) and obesity (15%) among KSRTC bus drivers, exceeding rates reported by Prabhu et al. and Sebastin in India, with 95.4% maintaining a lower-risk waist-hip ratio (<0.95), despite these factors being associated with cardiovascular health risks [30]. Night shift workers had 1.42 times higher odds of stress compared to day shift workers (OR=1.42, p=0.01), similar to Garbarino et al. (2018), who found increased stress among night shift workers in Italy [31].

This study, conducted within the organized KSRTC sector, offers valuable insights into occupational stress, with findings generalizable to similar settings due to the consistent working conditions. The use of a standardized questionnaire enhances reliability and comparability, while explicit inclusion and exclusion criteria minimize selection bias. However, limitations include the lack of standardized tools for assessing alcohol use and smoking and the observational design's inability to control for all confounders. The study recommends short-term interventions like regular health check-ups and mental health campaigns, alongside long-term strategies such as improved social security, ergonomic enhancements, and stress management training to reduce job strain and improve driver well-being.

## Conclusions

Our study reveals a significant prevalence of occupational stress among KSRTC bus drivers in Kolar, with stress levels strongly influenced by factors such as urban residence, divorce, tobacco chewing, chronic alcohol consumption, hypertension, a higher BMI, a high waist-hip ratio, and long working hours. These findings underscore the need for tailored stress management programs that address the specific lifestyle and job demands of bus drivers to improve their well-being and job performance. The present study also recommends preplacement examination and periodic health screening along with mental health screening among long-distance bus drivers.
